# Endorectal Ultrasound and Magnetic Resonance Imaging for Rectal Cancer Staging: A Modern Multimodality Approach

**DOI:** 10.3390/jcm10040641

**Published:** 2021-02-08

**Authors:** Alfonso Reginelli, Alfredo Clemente, Angelo Sangiovanni, Valerio Nardone, Francesco Selvaggi, Guido Sciaudone, Fortunato Ciardiello, Erika Martinelli, Roberto Grassi, Salvatore Cappabianca

**Affiliations:** 1 Radiology and Radiotherapy Unit, Department of Precision Medicine, University of Campania “L. Vanvitelli”, 80138 Naples, Italy; alfonsoreginelli@hotmail.com (A.R.); angsangiova@gmail.com (A.S.); roberto.grassi@unicampania.it (R.G.); salvatore.cappabianca@unicampania.it (S.C.); 2 Unit of Radiation Oncology, Ospedale del Mare, 80147 Naples, Italy; v.nardone@hotmail.it; 3 Colorectal Surgery, Department of Advanced Medical and Surgical Sciences, University of Campania “L. Vanvitelli”, 80138 Naples, Italy; francesco.selvaggi@unicampania.it (F.S.); guido.sciaudone@unicampania.it (G.S.); 4 Medical Oncology, Department of Precision Medicine, University of Campania “L. Vanvitelli”, 80138 Naples, Italy; fortunato.ciardiello@unicampania.it (F.C.); erikamartinelli75@yahoo.it (E.M.)

**Keywords:** rectal cancer, magnetic resonance imaging, endorectal ultrasonography, imaging, staging

## Abstract

Preoperative staging represents a crucial point for the management, type of surgery, and candidacy for neoadjuvant therapy in patient with rectal cancer. The most recent clinical guidelines in oncology recommend an accurate preoperative evaluation in order to address early and advanced tumors to different therapeutic options. In particular, potential pitfalls may occur in the assessment of T3 tumors, which represents the most common stage at diagnosis. The depth of tumor invasion is known to be an important prognostic factor in rectal carcinoma; as a consequence, the T3 imaging classification has a substantial importance for treatment strategy and patient survival. However, the differentiation between tumor invasion of perirectal fat and mesorectal desmoplastic reactions remains a main goal for radiologists. Magnetic resonance imaging (MRI) is actually considered as the best imaging modality for rectal cancer staging. Although the endorectal ultrasound (ERUS) is the preferred staging method for early tumors, it could also be useful in identifying perirectal fat invasion. Moreover, the addiction of diffusion weighted imaging (DWI) improves the diagnostic performance of MRI in rectal cancer staging by adding functional information about rectal tumor and adjacent mesorectal tissues. This study investigated the diagnostic performance of conventional MRI alone, in combination with the DWI technique and ERUS in order to assess the best diagnostic imaging combination for rectal cancer staging.

## 1. Introduction

Rectal cancer is one of the most common cancers worldwide and arises 30% of all colorectal tumors [1,2]. The diagnosis, treatment, and follow-up of patients with rectal cancer has considerably improved during the last years [3]. However, it is essential to obtain an adequate preoperative staging for their management. In particular, the assessment of cancer invasion through the rectal wall (T stage) remains the primary and most important factor for treatment planning in patients with rectal carcinoma [4]. Imaging key role has also been remarked by different oncologic guidelines, which recommend distinctive therapeutic approaches according to each T stage, in combination with other risk factors [5,6]. Patients with advanced T stage and mesorectal involvement have a high risk for local recurrence, and they are generally treated with long-term neoadjuvant chemoradiation therapy in order to increase the chance of a curative resection [5,6,7]. Particular attention should be paid in defining the T3 stage which arises 70% of all rectal tumors [8]. According to the AJCC/TNM classification, the T3 category arise tumors with invasion beyond the muscularis propria but not invading adjacent organs [9,10]. However, due to the high heterogeneous 5-year survival (ranging from 90% to 25%) [8], the T3 category has also been divided into four groups: T3a (<1 mm), T3b (1–5 mm), T3c (5–15 mm), and T3d (>15 mm), depending on the grade of invasion beyond the muscularis propria [10]. This subclassification is determined on the basis of a magnetic resonance imaging (MRI) evaluation and it is commonly adopted in the European guidelines for treatment recommendations [5,10,11]. Moreover, the latest European Guidelines of Oncology (ESMO) have recommended dedicated protocols for the management of T3 tumors, considering up-front surgery for the “early” (subgroups a and b) and short neoadjuvant therapy for the “advanced” T3 (subgroups c and d) [5]. These recent therapeutic strategies have further increased the role of preoperative imaging for the assessment of tumor stage, especially for borderline tumors at high risk of staging failure. MRI is the standard technique for rectal cancer staging [12,13,14,15]. An additional imaging technique, such as diffusion weighted imaging (DWI), has demonstrated an important contribution in the assessment of rectal cancer. Currently, DWI is the only noninvasive method able to detect diffuse movement of water molecules in living tissues, thus assessing tissue cellularity and integrity of cell membrane [16,17]. This technique has shown higher abilities in distinguishing tumor boundaries and in predicting tumor invasion than conventional MRI [11,13,15,16,17]. Endorectal ultrasonography (ERUS) is a reliable imaging method, able to evaluate rectal wall infiltration, especially for early tumors [4,18,19,20,21]. However, the imaging distinction between rectal cancer having a mesorectal fat invasion and desmoplastic reaction remains difficult and could mislead patients’ staging, causing important clinical implications [11,22,23,24]. Despite the recent technical improvements, all imaging modalities are still at risk of staging failure, with an overstaging rate reported up to 30% [22,23,24]. Numerous studies have reported on MRI and ERUS diagnostic performance for rectal cancer staging, but there are considerable differences in methodologic analysis, results and accuracy measures among them, particularly for T3 staging [4,22,24,25,26,27]. Moreover, none of the studies have been performed using three different imaging modalities in comparison and focused on a single stage of the disease. The purpose of our study was to evaluate the diagnostic performance of different imaging modalities (MRI, DWI, and ERUS) for the staging of rectal cancer. In particular, the imaging findings arising from each mentioned methods were analyzed for each clinical stage, then the results were compared with post-surgical findings.

## 2. Materials and Methods

This retrospective, single-center study was approved by the institutional review board, and written informed consent was obtained from all patients.

### 2.1. Patient Population

All patients with pathologically proven rectal cancer who underwent MRI and DWI evaluation and ERUS in our institution, were consecutively evaluated from June 2016 to December 2019. Only tumors with distal extension < 15 cm from the anal margin were included. Cancers up to 15 cm from the anal verge were considered as colonic tumors and were excluded from the analysis. Definitive rectal cancer staging was obtained through histological confirmation after surgical resection. All MRI, DWI, and ERUS findings were evaluated by comparing images and reports with the pathological findings, considered as the gold standard.

### 2.2. Imaging Interpretation

All imaging examinations were retrospectively interpreted by two blinded radiologists with subspecialty training (5 and 15 years) in gastrointestinal cancer imaging. The DWI images were obtained during the conventional MRI examinations, while ERUS were performed no more than 2 weeks later. Each reader was blinded to the MRI reports during ERUS examinations.

### 2.3. Magnetic Resonance Imaging

All MRI examinations were performed with a 1.5 T scanner (Magnetom Symphony, Siemens Medical System, Erlangen, Germany) using an eight-channel phased array coil before and after the intravenous injection of paramagnetic contrast medium (Gadobutrol, 0.1 mL/kg, Gadovist^®^, Schering AG, Berlin, Germany) volumes ranging from 5 to 9 mL and injection rates from 1.5 to 2.0 mL/s. The imaging protocol was chosen following the European Society of Gastrointestinal Abdominal Radiology (ESGAR) recommendations [28]. It consisted of a standard thin section (3 mm) axial T2-weighted fast spin-echo sequence performed in a plane orthogonal to the tumor followed by high-spatial-resolution T2-weighted fast spin-echo coronal images and high-spatial resolution sagittal T2-weighted images. Axial gradient-echo T1-weighted sequences with fat suppression were performed before and after the i.v. injection of contrast media. The conventional MRI protocol was completed by DWI acquisitions in the axial plane (b-values 0, 500, 1000) and relative apparent diffusion coefficient (ADC) maps. Rectal lesions and lymph nodes were assessed according to the established imaging criteria [29,30,31,32]. For each patient, a tumor stage was determined according to the AJCC/TNM classification [10].

### 2.4. ERUS

ERUS examinations were performed using a 360° ultrasound probe (High-Vision, Hitachi Medical Corporation, Kashiva, Japan) at a frequency of 10–13 MHz. The evaluations were carried out initially in two-dimensions, followed by three-dimensional (3D) sequential acquisitions throughout the length of the rectal canal. The images were automatically reconstructed as a cube and worked on later. 3D ERUS images were acquired only once and recorded in video format in order to be reviewed later by the second operator. In particular, this technique allowed a post examination analysis of the 3D-ERUS scan in coronal, sagittal, or axial planes as deemed necessary. All examinations were performed in lateral decubitus position starting from the upper third of the rectum to the anal verge. The US staging was made according to the standard of references [33]: stage uT1 tumors were confined to the mucosa and submucosa; uT2 tumors invaded the muscularis propria but not the rectal wall; uT3 tumors penetrated the rectal wall and the perirectal fat; uT4 tumors invaded the nearby pelvic structures.

### 2.5. Statistical Analysis

The statistical analysis was carried out considering the results obtained from the single imaging techniques. For each imaging modality were evaluated sensitivity (SE), specificity (SP), positive predictive value (PPV), and negative predictive value (VPN) using the pathology result as the standard of reference. The degree of agreement between the observers was measured by both percentage agreement of the total number of observations, considering the total number of times in which the observers agreed, which was divided by the total number of readings/classifications made, and by calculating Cohen’s kappa (κ) coefficient. Perfect agreement was evident when Cohen’s kappa equaled 1; a value of Cohen’s kappa equal to zero suggested that the agreement was no better than that which would be obtained by chance alone. All the analyses were carried out using SPSS 20.0 software (SPSS Inc., Chicago, IL, USA).

## 3. Results

**Figure 1:**
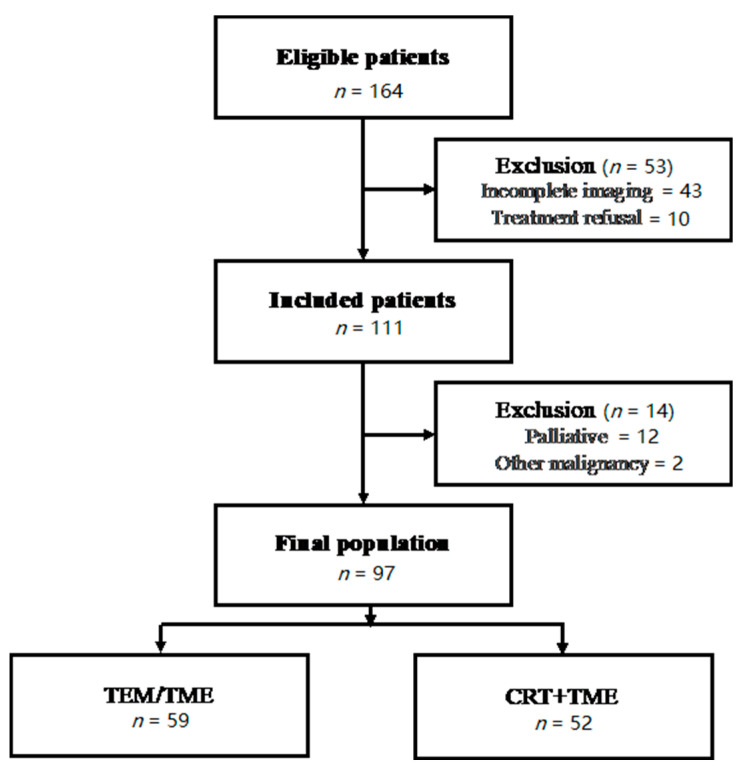
The diagram summarizes the exclusion criteria adopted to obtain a homogeneous population in relation to the type of treatment received. TEM: transanal endoscopic microsurgery; TME: total mesorectal excision; CRT: chemo-radiotherapy.

The study population included 164 patients. Ninety-seven were considered suitable in terms of homogeneity of the sample in relation to the characteristics analyzed. Sixty-two were male and 35 were females with a median age of 66 years (range 34–84). The selection criteria of final population and the inclusion and exclusion criteria are summarized in Figure 1. Pathological staging (pT) of the primary tumor, obtained after surgical examination identified: pT1, 3 patients, pT2, 22 patients, pT3, 61 patients, and pT4, 11 patients. Lymph node involvement (pN +) was present in 65 patients out of 97. The results obtained by each imaging modality in tumor stage assessment are compared with pathological findings in Table 1, Table 2 and Table 3. The diagnostic performances of MRI, DWI, and ERUS were evaluated for each stage, and they are summarized in Table 2. The degree of agreement between the two observers is shown in Table 3. Both the percentage agreement of the total number of observations and the κ coefficient showed excellent agreement among the observers when considering different imaging methods.

## 4. Discussion

**Figure 2:**
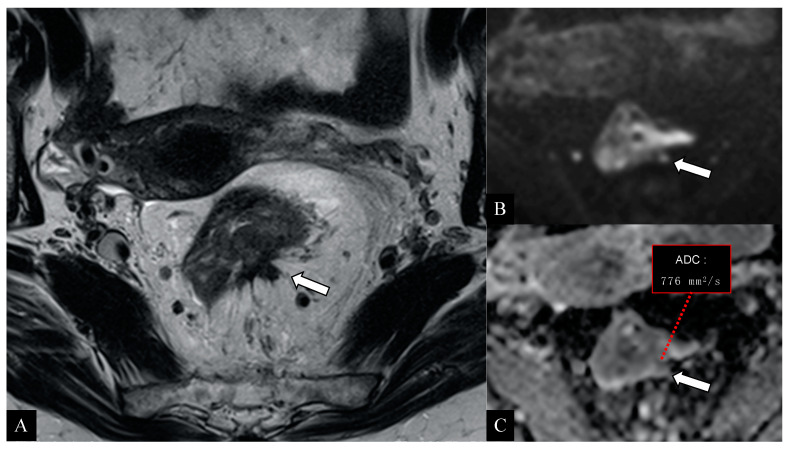
58-year-old woman with histologically proven rectal adenocarcinoma. High-resolution axial T2 image (**A**) demonstrates a rectal neoplasm associated with a markedly hypointense tissue with irregular borders and retracting appearance (arrow) suggestive of an inflammatory/desmoplastic reaction in the contiguous mesorectal fat (T2 stage). The diffusion-weighted axial images (**B**) with relative apparent diffusion coefficient (ADC) map (**C**) reveal a hypercellular tissue with marked diffusivity restriction (low ADC values; inset) suggestive for neoplastic infiltration of the mesorectum > 5 mm (arrow). The histological examination confirmed the mesorectal infiltration and the preoperative staging (stage: T3c).

**Figure 3:**
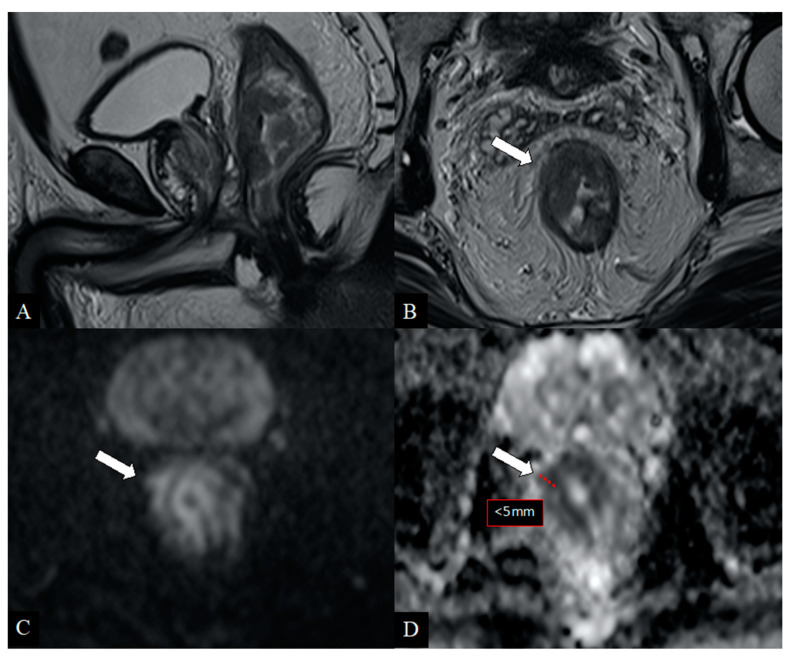
48-year-old patient with rectal cancer. Sagittal (**A**) and axial (**B**) T2-weighted images show heterogeneous tissue involving the right hemicircumference of the lower rectum with a focal breakpoint at 10 o‘clock (arrow) doubtful due to wall infiltration (T2/T3 stage). The diffusion-weighted axial images (**C**) with relative ADC map (**D**) show a clear extraparietal component < 5 mm (arrows) with diffusion restriction and very low ADC map values (pathological hypercellular tissue). Histological examination confirmed the mesorectal extension of the disease (stage T3b).

**Figure 4:**
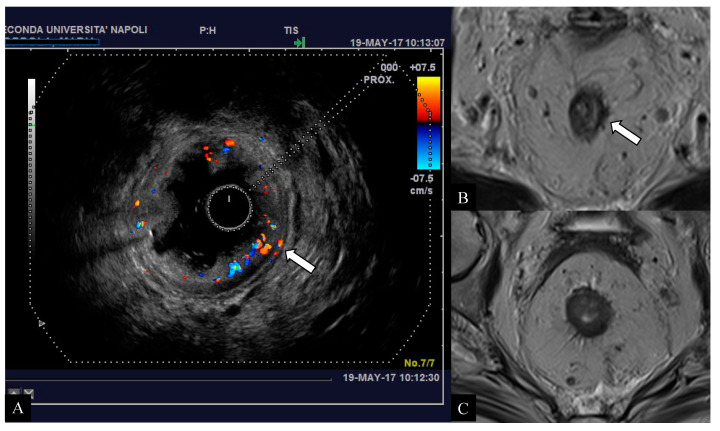
Early-stage tumor affecting the middle rectum in a 64-year-old woman. Endorectal ultrasound (ERUS) scan (**A**) demonstrates the presence of pathological rectal tissue confined to the left submucosal wall with preserved definition of the hypoechoic layer of muscolaris propria (arrow) referable to an initial clinical stage (T2) of the disease. The axial T2-weighted images (**B**,**C**) obtained with magnetic resonance imaging (MRI) raised the suspicion of extra-parietal infiltration due to evidence of spiculation in the perivisceral mesorectal tissue (arrow). The histological examination confirmed the desmoplastic nature of the tissue with the presence of granulocytes and eosinophils, suggestive for a reactive/inflammatory tissue.

**Figure 5:**
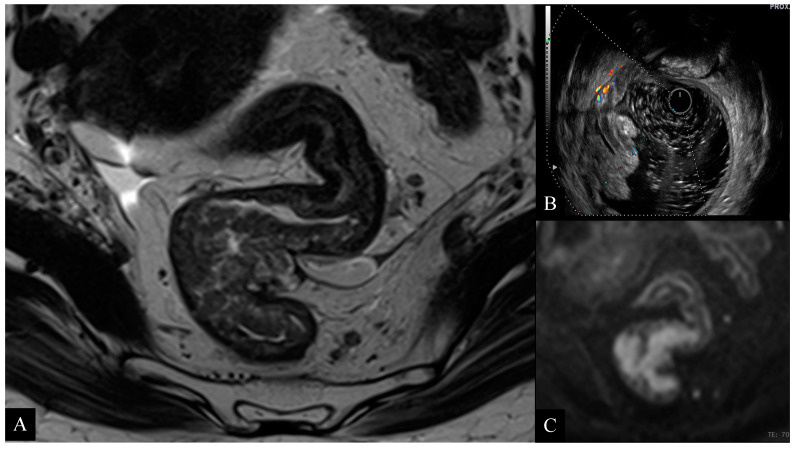
67-year-old woman with high rectal cancer. Axial T2-weighted image (**A**) obtained with MRI shows a large circumferential tumor infiltrating the muscolaris propria layer and close to the anterior peritoneal reflection. ERUS scan (**B**) and axial diffusion weighted imaging (DWI) (**C**) images confirm the presence of a tumor infiltrating the rectal wall with focal invasion to the perirectal fat. Pathology examination confirmed the presence of a T3b tumor.

Preoperative staging represents a key point in the management of patients with rectal cancer. An accurate preoperative assessment is deemed decisive for an appropriate treatment choice and improves patient’s prognosis, reducing the probability of relapse [30,31]. For many years, the standard clinical approach has been based on neoadjuvant therapy before surgery, especially for patients with rectal cancer at intermediate risk, thus accepting excessive treatment rather than an insufficient one. However, this strategy has exposed these patients to many side effects of chemoradiation (i.e., sexual, urinary, defecatory disorders) with rather modest improvement in the overall survival rates compared to those not exposed [25,34]. In particular, anterior resection syndrome (around 50–90% of patients undergoing total mesorectal excision (TME)) would manifest its symptoms with higher severity (i.e., urgency, incontinence, diarrhea) in patients previously treated with radiotherapy (regardless of protocol used) resulting in a worsening of patient’s quality of life [25]. Furthermore, it has been demonstrated that the neoadjuvant treatment in these patients does not guarantee a significant local control of the disease more than the primary surgery alone [25,35]. For these reasons, the European Society of Medical Oncology (ESMO) recommends the neoadjuvant treatment prior to surgery only in cases of patients with advanced T stage (T3c–T4) and lymph node involvement (N1–N2), resulting in an increased number of patients eligible for surgery alone [5]. As a consequence, adequate preoperative imaging has increased in importance. However, it still conceals many difficulties in clinical practice, especially in defining borderline tumors or subgroups. The analysis of our study population confirmed that most rectal cancers are clinically diagnosed at the T3 stage (62.8%) [8]. In our series, preoperative MRI based on conventional high-resolution T2 sequences showed sensitivity and specificity rates between 66.7–90.9% and 87.5–98.9%, respectively; these results are in line with previous experiences [22,36,37,38]. On the other side, sensitivity in MRI resulted lower (87.5%) in T3 cases, confirming the well-known limits described in the literature [29]. In particular, the differentiation between tumor infiltration into the mesorectal fat and desmoplastic tissues reaction still remains a diagnostic challenge. Many papers have highlighted this MRI-related issue, which tends to overestimate the initial T3 tumors (with infiltration < 5 mm of the mesorectum) in most cases [11,23,24,25,29,39,40,41]. In our experience, the early T3 tumors (subgroups a,b) have been overstaged (c,d categories or higher) in only 6 cases (6/61; 9.8%); sensibly lower than the average 19%, as reported in the literature by using MRI [25]. All overstaged tumors presented spicules in the mesorectal fat, then considered as tumoral invasion by both the observers. As is well-known, MRI is sometimes unable to differentiate between desmoplastic reaction from extramural tumor invasion when the infiltration does not have a nodular configuration or presents low signal intensity on T2 images [23]. The preoperative staging performed with MRI and DWI technique combined has provided interesting results in our study. In particular, in all tumor stages we obtained higher sensitivity and specificity rates than conventional MRI. Specifically, the sensitivity rates were 100% for T1 and T4 stages and 91.9% for the T3 stage. Our results are in line with previous series [24,42,43,44], even offering additional interpretations. In fact, the authors reported a higher diagnostic accuracy of DWI than conventional MRI technique but did not provide comparative results per tumor stage, especially for T3 cancers. Surprisingly, in our experience, the additional use of DWI improved the sensitivity of conventional MRI in both T3 and other tumor stages, reducing the cases of overestimation (for T3: 3/61; 4.9%) (Figure 2 and Figure 3). These results underline the novel and important contribution that this technique could also provide for tumor staging. In particular, the opportunity to differentiate hypercellular tissues (neoplasm vs. inflammation) through the evaluation of ADC map represents a modern strategy that can assist in tumor depiction [15,16]. The evaluation of ERUS capabilities confirmed the excellent diagnostic value of this method in early rectal cancer assessment (stage T1) in our cases as well. Conversely, the lowest sensitivity rate occurred for T2 tumors (76.6%), which represents the real weakness of this modality, as just reported in the literature [20]. Our experience, in alignment to Kav T. et al. [4], has showed its greatest difficulty in characterizing transmural tumor extension, leading to a consequent T2 overstaging. Converesely, better results were obtained for the assessment of early T3 tumors (sensitivity 95.1%), in line with previous series [18,20,22]. Moreover, the high percentage of agreement between the two operators (88.2%) suggests a good reliability of our findings. The most interesting results of the present study were obtained combining diagnostic features of different imaging modalities and considering the multimodal/multiparametric approach as a “model of choice” for rectal cancer staging (Figure 4 and Figure 5). The combined use of MRI, DWI, and ERUS reached the highest sensitivity and specificity values in all tumor stages, in particular for T3 cancers (95.1% and 99.8%, respectively). According to our knowledge, several studies have evaluated the diagnostic performance of each imaging techniques, but none of them have offered a cumulative analysis arising from a multimodal approach stratified for single stage [22,24,45,46]. Moreover, our results demonstrated a good concordance (82%) between multimodal imaging findings and pathological results against 63–69% reported in the literature [22,27,40]. As a consequence, we observed a lower number of overstaged patients (9.8%), in contrast to average reported rates ranging from 15% to 24% [25,45,46,47,48,49,50]. Moreover, the percentage of understaged patients was 8.2% in our study population, while it ranged from 12 to 24% in previous studies [22,25,40]. Therefore, a multimodal imaging approach based on MRI, DWI, and ERUS could reduce staging failures and avoid over- and understaging problems in patients with rectal cancer. Lastly, this method could also allow greater benefits in terms of patient’s outcome and in quality of life by receiving proper treatment.

The limitations of the present study were: (1) the small number of patients (nr. 97) in relation to the population eligible for multimodal staging of rectal pathology (164), largely due to the lack of postoperative histological results and incomplete imaging work-up; (2) the nature of the retrospective work, which did not allow a prospective selection of the analyzed variables; (3) the lack of an assessment of the 5-year survival of analyzed patients.

## 5. Conclusions

The clinical relevance of a preoperative staging requires a dedicated imaging approach in patients with rectal cancer. The DWI technique improves the diagnostic performance of conventional MRI alone for rectal cancer staging. A significant reduction in staging failure mainly related to overestimation could be obtained by a multimodal imaging approach based on MRI, DWI, and ERUS. However, further analyses are required to confirm the role of this multimodal approach in clinical practice.

## Figures and Tables

**Table 1:** Preoperative staging was obtained from different imaging modalities and compared with the post-surgical pathology results. The radiological assessment for each tumor stage obtained with MRI (**a**), DWI (**b**), and ERUS (**c**) were evaluated according the AJCC/TNM 8th Ed. [10].

***a***					
	**Pathology Stage**				
**MRI-Stage**	**T1**	**T2**	**T3**	**T4**	**Total**
T1	2	0	0	0	2
T2	1	16	0	0	17
T3	0	6	58	1	65
T4	0	0	3	10	13
Total	3	22	61	11	97
***b***					
	**Pathology Stage**				
**MRI+DWI-Stage**	**T1**	**T2**	**T3**	**T4**	**Total**
T1	3	0	0	0	3
T2	0	19	0	0	19
T3	0	3	59	0	62
T4	0	0	2	10	13
Total	3	22	61	11	97
***c***					
	**Pathology Stage**				
**ERUS-Stage**	**T1**	**T2**	**T3**	**T4**	**Total**
T1	3	0	0	0	3
T2	0	20	3	0	23
T3	0	2	58	1	61
T4	0	0	0	10	10
Total	3	22	61	11	97

**Table 2:** Diagnostic performance of MRI, DWI, and ERUS for each tumor stage were evaluated considering the post-surgical results as the gold standard (CI: 95%).

STAGE	MRI					MRI + DWI					ERUS				
	N	SE	SP	VPP	VPN	N	SE	SP	VPP	VPN	N	SE	SP	VPP	VPN
**T1**	2	66.7	98.1	66.7	98.9	3	100.0	100.0	100.0	100.0	3	100.0	100.0	100.0	100.0
**T2**	17	76.2	94.2	76.2	94.1	19	86.4	96.3	86.4	96.3	23	81.8	95.1	81.8	95.1
**T3**	65	82.1	87.5	90.2	77.8	62	91.9	90.9	93.4	88.8	61	95.1	100.0	100.0	92.3
**T4**	13	90.9	98.9	90.9	98.9	13	100.0	100.0	100.0	100.0	10	90.1	98.9	90.9	98.9

**Table 3:** Degree of agreement between the two observers in relation to the differences in imaging techniques used.

Agreement	MRI		ERUS
	Conventional	DWI	
Agreement (%)	88.5	92.7	88.5
Coefficient kappa Cohen (κ)	0.759	0.845	0.762

## Data Availability

Data are available upon request to the corresponding author.
